# The genome sequence of the furry-claspered furrow bee, *Lasioglossum lativentre *(Schenck, 1853)

**DOI:** 10.12688/wellcomeopenres.17706.1

**Published:** 2022-02-15

**Authors:** Steven Falk, Joseph Monks, University of Oxford and Wytham Woods Genome Acquisition Lab, Darwin Tree of Life Barcoding collective, Wellcome Sanger Institute Tree of Life programme, Wellcome Sanger Institute Scientific Operations: DNA Pipelines collective, Tree of Life Core Informatics collective, Darwin Tree of Life Consortium

**Affiliations:** 1 Independent Researcher, Kenilworth, UK; 2 Department of Life Sciences, Natural History Museum, London, UK

**Keywords:** Lasioglossum lativentre, furry-claspered furrow bee, genome sequence, chromosomal, Hymenoptera

## Abstract

We present a genome assembly from an individual male *Lasioglossum lativentre *(the furry-claspered furrow bee; Arthropoda; Insecta; Hymenoptera; Halictidae). The genome sequence is 479 megabases in span. The majority of the assembly (75.22%) is scaffolded into 14 chromosomal pseudomolecules. The mitochondrial genome was also assembled, and is 15.3 kilobases in length.

## Species taxonomy

Eukaryota; Metazoa; Ecdysozoa; Arthropoda; Hexapoda; Insecta; Pterygota; Neoptera; Endopterygota; Hymenoptera; Apocrita; Aculeata; Apoidea; Anthophila; Halictidae; Halictinae; Halictini; *Lasioglossum (Lasioglossum) lativentre* (Schenck, 1853) (NCBI:txid2795680).

## Background

*Lasioglossum (Lasioglossum) lativentre* (furry-claspered furrow bee) is a solitary, ground nesting bee found throughout the western Palearctic from the UK to Iran. In the UK the genus *Lasioglossum* Curtis is represented by 32 species, but worldwide there are at least 1,700 species. *L. lativentre* is common in lowland England up to Yorkshire and southern Wales. The species shows a particular association with plant species of the family Asteraceae ( Falk, 2015). The species is found along woodland edges but can also occur in gardens and other grassland habitats. Females emerge first in March with males appearing later in June. The cleptoparasites *Sphecodes ephippius* (Linnaeus) and *S. puncticeps* Thomson use *L. lativentre* as their host.

## Genome sequence report

**Figure 1.**
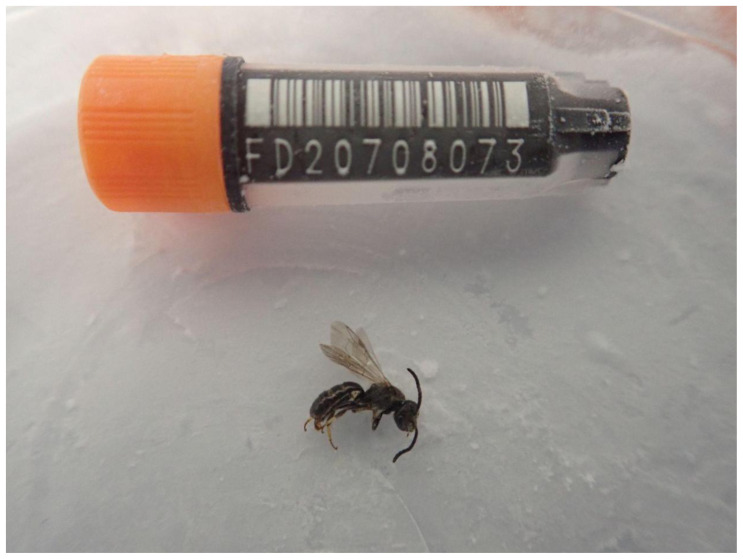
Image of the iyLasLatv2 specimen taken during preservation and processing.

The genome was sequenced from a single male *L. lativentre* ( Figure 1) collected from Wytham Woods, Oxfordshire (biological vice-county: Berkshire), UK (latitude 51.769, longitude -1.339). A total of 34-fold coverage in Pacific Biosciences single-molecule long reads and 54-fold coverage in 10X Genomics read clouds were generated. Primary assembly contigs were scaffolded with chromosome conformation Hi-C data. Manual assembly curation corrected 50 missing/misjoins and removed 14 haplotypic duplication, reducing the scaffold number by 2.97%, and increasing the scaffold N50 by 70.83%.

**Figure 2.**
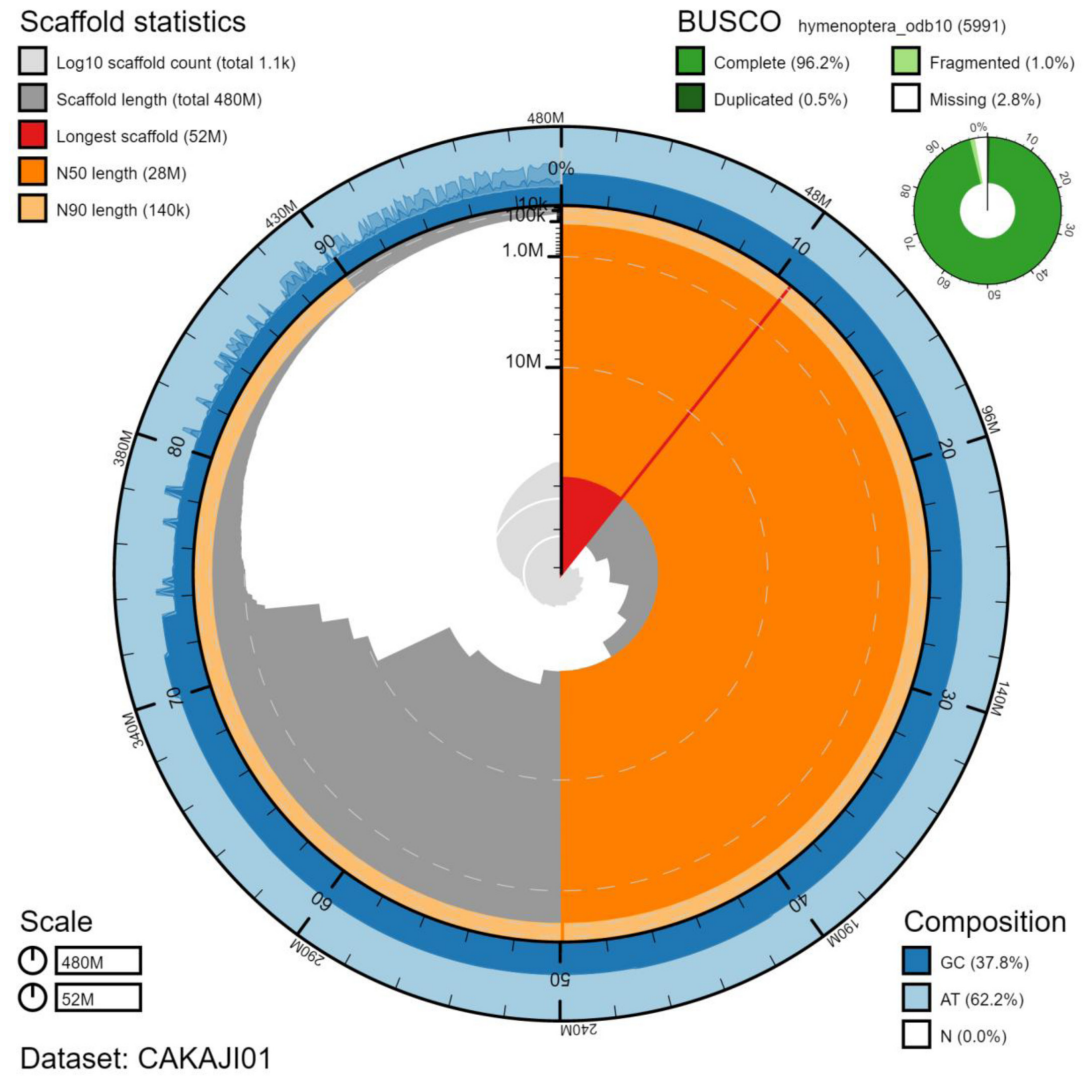
Genome assembly of *Lasioglossum lativentre*, iyLasLatv2.1: metrics. The BlobToolKit Snailplot shows N50 metrics and BUSCO gene completeness. The main plot is divided into 1,000 size-ordered bins around the circumference with each bin representing 0.1% of the 478,951,010 bp assembly. The distribution of chromosome lengths is shown in dark grey with the plot radius scaled to the longest chromosome present in the assembly (51,674,092 bp, shown in red). Orange and pale-orange arcs show the N50 and N90 chromosome lengths (27,713,800 and 136,982 bp), respectively. The pale grey spiral shows the cumulative chromosome count on a log scale with white scale lines showing successive orders of magnitude. The blue and pale-blue area around the outside of the plot shows the distribution of GC, AT and N percentages in the same bins as the inner plot. A summary of complete, fragmented, duplicated and missing BUSCO genes in the hymenoptera_odb10 set is shown in the top right. An interactive version of this figure is available at https://blobtoolkit.genomehubs.org/view/iyLasLatv2.1/dataset/CAKAJI01/snail.

The final assembly has a total length of 479 Mb in 1143 sequence scaffolds with a scaffold N50 of 27.7 Mb ( Table 1). Of the assembly sequence, 75.22% was assigned to 14 chromosomal-level scaffolds (numbered by sequence length) ( Figure 2– Figure 5; Table 2). The assembly has a BUSCO v5.1.2 ( Manni *et al*., 2021) completeness of 96.2% (single 95.6%, duplicated 0.5%) using the hymenoptera_odb10 reference set (n=5991). While not fully phased, the assembly deposited is of one haplotype. Contigs corresponding to the second haplotype have also been deposited.

**Table 1.** Genome data for *Lasioglossum lativentre*, iyLasLatv2.1.

*Project accession data*	
Assembly identifier	iyLasLatv2.1
Species	*Lasioglossum lativentre*
Specimen	iyLasLatv2 (male, genome assembly); iyLasLatv1 (female)
NCBI taxonomy ID	NCBI:txid88531
BioProject	PRJEB46299
BioSample ID	SAMEA7746765
Isolate information	Whole organisms
*Raw data accessions*	
PacificBiosciences SEQUEL II	ERR6939226
10X Genomics Illumina	ERR6688421-ERR6688424
Hi-C Illumina	ERR6688420
*Genome assembly*	
Assembly accession	GCA_916610255.1
*Accession of alternate haplotype*	GCA_916610185.1
Span (Mb)	479
Number of contigs	1430
Contig N50 length (Mb)	4.1
Number of scaffolds	1143
Scaffold N50 length (Mb)	27.7
Longest scaffold (Mb)	51.7
BUSCO * genome score	C:96.2%[S:95.6%,D:0.5%],F:1.0%,M:2.8%,n:5991

*BUSCO scores based on the hymenoptera_odb10 BUSCO set using v5.1.2. C= complete [S= single copy, D=duplicated], F=fragmented, M=missing, n=number of orthologues in comparison. A full set of BUSCO scores is available at https://blobtoolkit.genomehubs.org/view/iyLasLatv2.1/dataset/CAKAJI01/busco.

**Figure 3.**
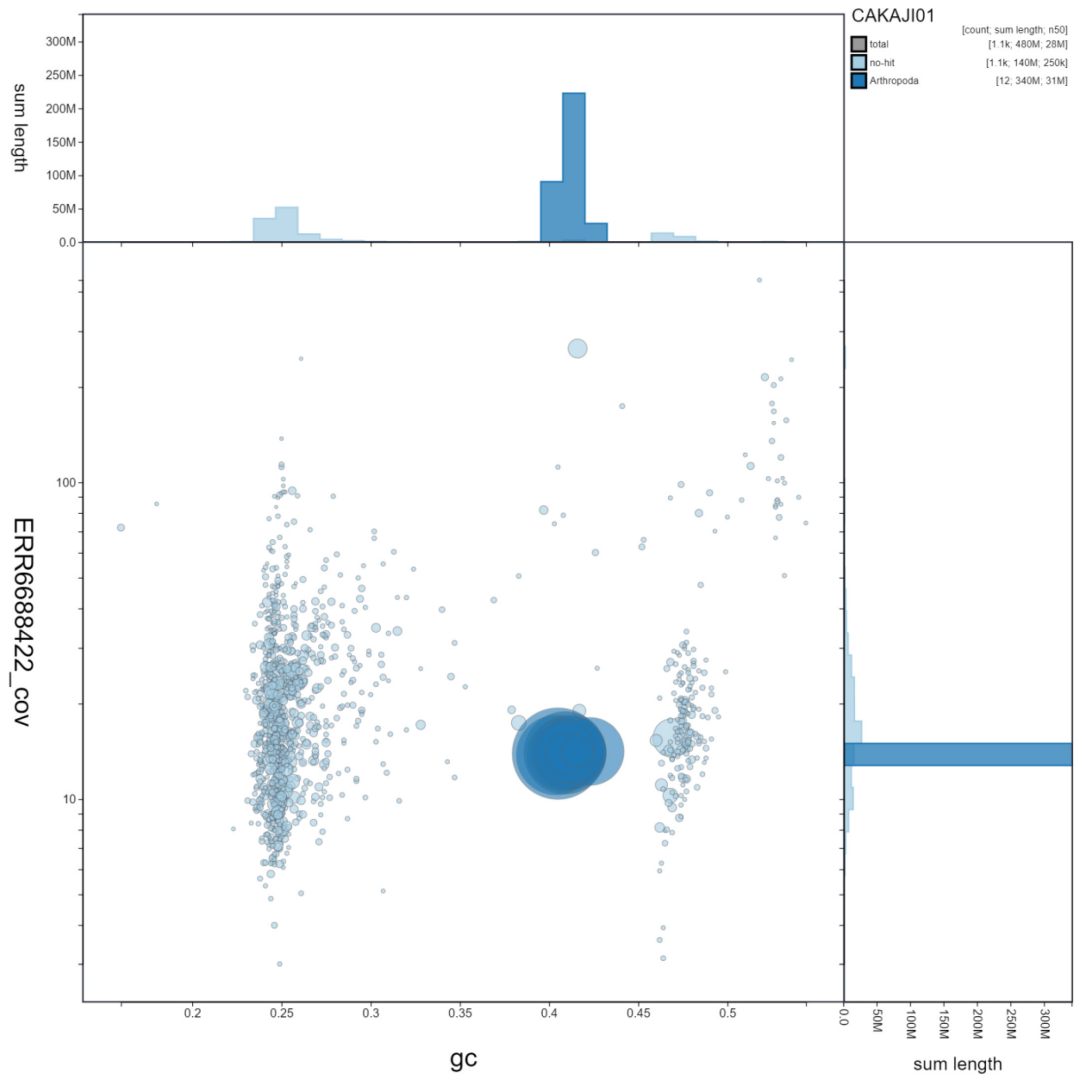
Genome assembly of *Lasioglossum lativentre*, iyLasLatv2.1. GC coverage. BlobToolKit GC-coverage plot. Scaffolds are coloured by phylum. Circles are sized in proportion to scaffold length. Histograms show the distribution of scaffold length sum along each axis. An interactive version of this figure is available at https://blobtoolkit.genomehubs.org/view/iyLasLatv2.1/dataset/CAKAJI01/blob.

**Figure 4.**
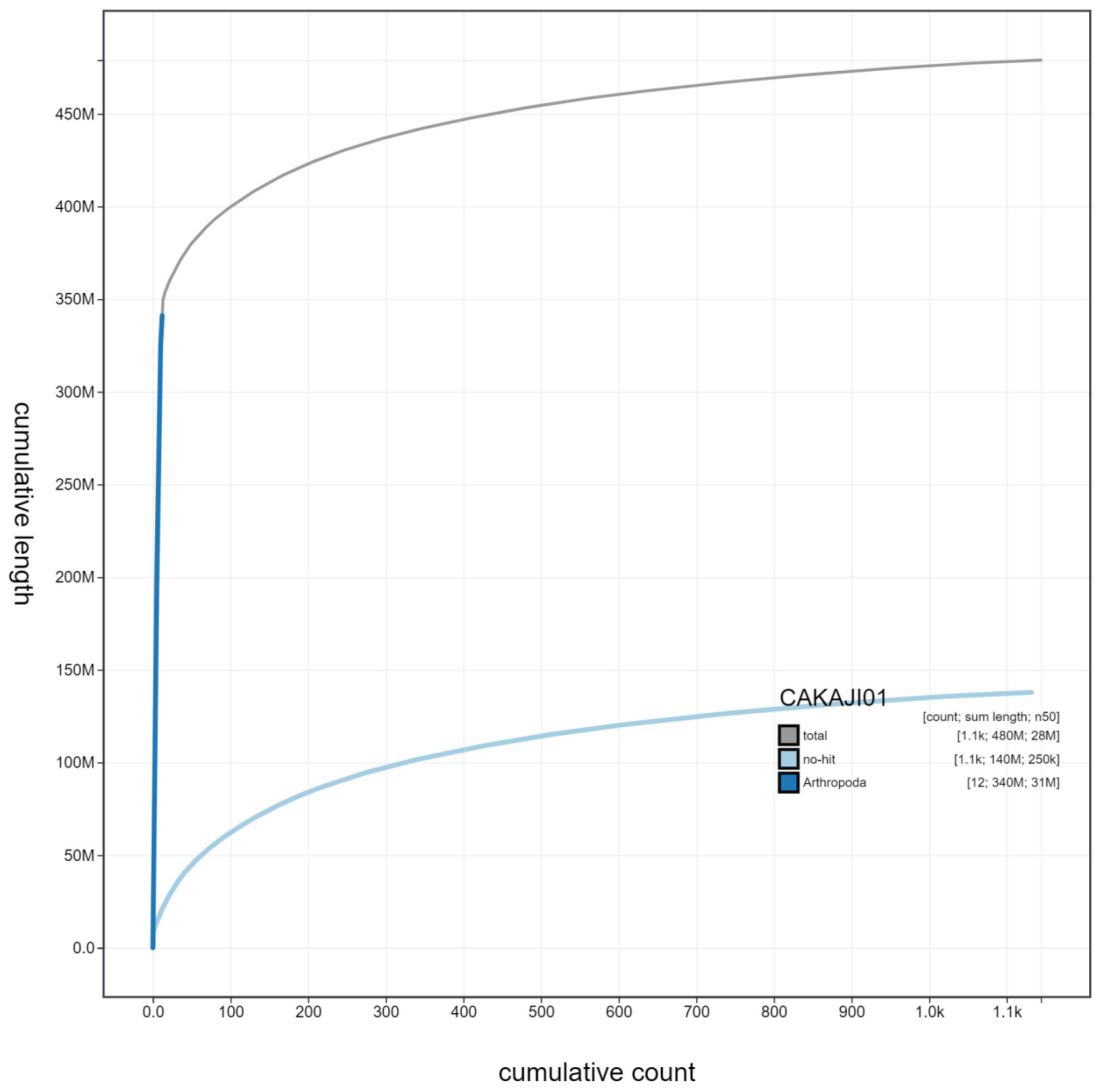
Genome assembly of *Lasioglossum lativentre*, iyLasLatv2.1: cumulative sequence. BlobToolKit cumulative sequence plot. The grey line shows cumulative length for all scaffolds. Coloured lines show cumulative lengths of scaffolds assigned to each phylum using the buscogenes taxrule. An interactive version of this figure is available at https://blobtoolkit.genomehubs.org/view/iyLasLatv2.1/dataset/CAKAJI01/cumulative.

**Figure 5.**
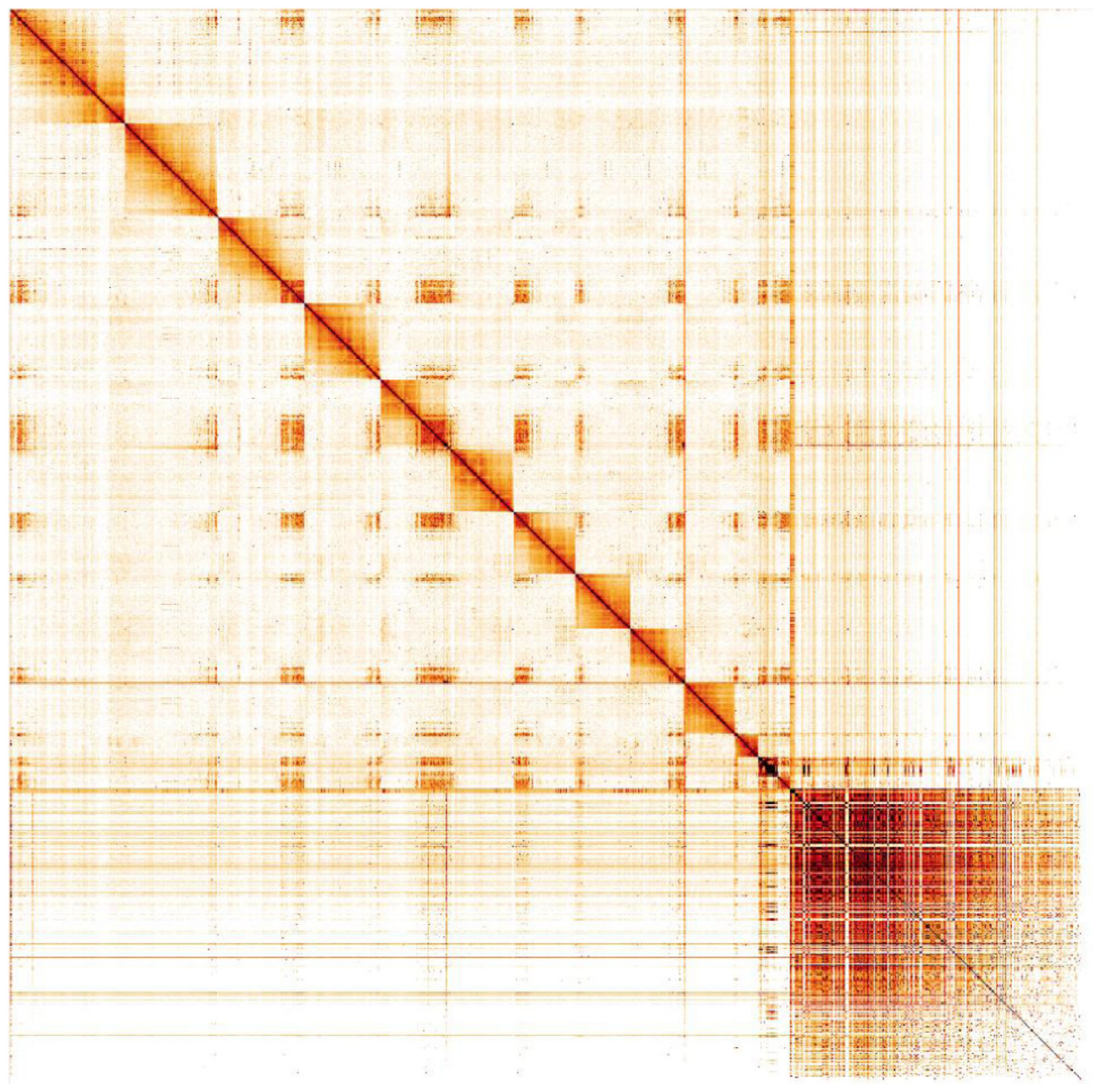
Genome assembly of *Lasioglossum lativentre*, iyLasLatv2.1: Hi-C contact map. Hi-C contact map of the iyLasLatv2.1 assembly, visualised in HiGlass. Chromosomes are shown in size order from left to right and top to bottom.

**Table 2.** Chromosomal pseudomolecules in the genome assembly of *Lasioglossum lativentre*, iyLasLatv2.1.

INSDC accession	Chromosome	Size (Mb)	GC%
OU744355.1	1	51.67	40.5
OU744356.1	2	41.59	40.9
OU744357.1	3	38.71	40.4
OU744358.1	4	34.03	41.1
OU744359.1	5	31.46	40.8
OU744360.1	6	27.89	42.3
OU744361.1	7	27.71	41.1
OU744362.1	8	24.76	41.4
OU744363.1	9	24.66	41.3
OU744364.1	10	22.59	40.9
OU744365.1	11	8.53	46.9
OU744366.1	12	10.26	41.0
OU744367.1	13	5.79	41.6
OU744368.1	14	1.81	41.7
OU744369.1	MT	0.02	18.5
-	Unplaced	127.46	28.5

## Methods

### Sample acquisition and DNA extraction

A male (iyLasLatv2) and a female (iyLasLatv1) *L. lativentre* were collected from Wytham Woods, Oxfordshire (biological vice-county: Berkshire), UK (latitude 51.769, longitude -1.339) by Steven Falk, Independent Researcher, using a net. The samples were identified by the same individual and snap-frozen on dry ice.

DNA was extracted at the Tree of Life laboratory, Wellcome Sanger Institute. The iyLasLatv2 sample was weighed and dissected on dry ice with tissue set aside for Hi-C sequencing. Whole organism tissue was disrupted using a Nippi Powermasher fitted with a BioMasher pestle. Fragment size analysis of 0.01-0.5 ng of DNA was then performed using an Agilent FemtoPulse. High molecular weight (HMW) DNA was extracted using the Qiagen MagAttract HMW DNA extraction kit. Low molecular weight DNA was removed from a 200-ng aliquot of extracted DNA using 0.8X AMpure XP purification kit prior to 10X Chromium sequencing; a minimum of 50 ng DNA was submitted for 10X sequencing. HMW DNA was sheared into an average fragment size between 12-20 kb in a Megaruptor 3 system with speed setting 30. Sheared DNA was purified by solid-phase reversible immobilisation using AMPure PB beads with a 1.8X ratio of beads to sample to remove the shorter fragments and concentrate the DNA sample. The concentration of the sheared and purified DNA was assessed using a Nanodrop spectrophotometer and Qubit Fluorometer and Qubit dsDNA High Sensitivity Assay kit. Fragment size distribution was evaluated by running the sample on the FemtoPulse system.

### Sequencing

Pacific Biosciences HiFi circular consensus and 10X Genomics read cloud sequencing libraries were constructed according to the manufacturers’ instructions. Sequencing was performed by the Scientific Operations core at the Wellcome Sanger Institute on Pacific Biosciences SEQUEL II and Illumina NovaSeq 6000 instruments. Hi-C data were generated from whole organism tissue of iyLasLatv1 using the Arima v2.0 kit and sequenced on an Illumina NovaSeq 6000 instrument.

### Genome assembly

Assembly was carried out with Hifiasm ( Cheng *et al*., 2021). Haplotypic duplication was identified and removed with purge_dups ( Guan *et al*., 2020). Scaffolding with Hi-C data ( Rao *et al*., 2014) was carried out with SALSA2 ( Ghurye *et al*., 2019). The Hi-C scaffolded assembly was polished with the 10X Genomics Illumina data by aligning to the assembly with longranger align, calling variants with freebayes ( Garrison & Marth, 2012). One round of the Illumina polishing was applied. The mitochondrial genome was assembled with MitoHiFi ( Uliano-Silva *et al*., 2021), which performed annotation using MitoFinder ( Allio *et al*., 2020). The assembly was checked for contamination as described previously ( Howe *et al*., 2021). Manual curation ( Howe *et al*., 2021) was performed using HiGlass ( Kerpedjiev *et al*., 2018) and Pretext. The genome was analysed within the BlobToolKit environment ( Challis *et al*., 2020). Table 3 contains a list of all software tool versions used, where appropriate.

**Table 3.** Software tools used.

Software tool	Version	Source
Hifiasm	0.15.2	Cheng *et al*., 2021
purge_dups	1.2.3	Guan *et al*., 2020
SALSA2	2.2	Ghurye *et al*., 2019
longranger align	2.2.2	https://support.10xgenomics.com/genome-exome/software/pipelines/latest/advanced/other-pipelines
freebayes	v1.3.1-17-gaa2ace8	Garrison & Marth, 2012
MitoHiFi	2	Uliano-Silva *et al*., 2021
HiGlass	1.11.6	Kerpedjiev *et al*., 2018
PretextView	0.2.x	https://github.com/wtsi-hpag/PretextView
BlobToolKit	2.6.4	Challis *et al*., 2020

### Ethics/compliance issues

The materials that have contributed to this genome note have been supplied by a Darwin Tree of Life Partner. The submission of materials by a Darwin Tree of Life Partner is subject to the Darwin Tree of Life Project Sampling Code of Practice. By agreeing with and signing up to the Sampling Code of Practice, the Darwin Tree of Life Partner agrees they will meet the legal and ethical requirements and standards set out within this document in respect of all samples acquired for, and supplied to, the Darwin Tree of Life Project. Each transfer of samples is further undertaken according to a Research Collaboration Agreement or Material Transfer Agreement entered into by the Darwin Tree of Life Partner, Genome Research Limited (operating as the Wellcome Sanger Institute), and in some circumstances other Darwin Tree of Life collaborators.

## Data availability

European Nucleotide Archive: Lasioglossum lativentre (furry-claspered furrow bee). Accession number PRJEB46299; https://identifiers.org/ena.embl/PRJEB46299.

The genome sequence is released openly for reuse. The *L. lativentre* genome sequencing initiative is part of the Darwin Tree of Life (DToL) project. All raw sequence data and the assembly have been deposited in INSDC databases. The genome will be annotated and presented through the Ensembl pipeline at the European Bioinformatics Institute. Raw data and assembly accession identifiers are reported in Table 1.

## Author information

Members of the University of Oxford and Wytham Woods Genome Acquisition Lab are listed here: https://doi.org/10.5281/zenodo.5746938.

Members of the Darwin Tree of Life Barcoding collective are listed here: https://doi.org/10.5281/zenodo.5744972.

Members of the Wellcome Sanger Institute Tree of Life programme are listed here: https://doi.org/10.5281/zenodo.5744840.

Members of Wellcome Sanger Institute Scientific Operations: DNA Pipelines collective are listed here: https://doi.org/10.5281/zenodo.5746904.

Members of the Tree of Life Core Informatics collective are listed here: https://doi.org10.5281/zenodo.5743293.

Members of the Darwin Tree of Life Consortium are listed here: https://doi.org/10.5281/zenodo.5638618.
