# Supplementary material for: The genome sequence of the furry-claspered furrow bee, Lasioglossum lativentre (Schenck, 1853)
Source: Wellcome Open Res. 2022 Feb 15;7:57. [Version 1] doi: 10.12688/wellcomeopenres.17706.1 (PMC9971656; doi:10.12688/wellcomeopenres.17706.1)
Supplement: Peer review history

# Peer review: Reviewer response for version 1

**DOI:** 10.21956/wellcomeopenres.19593.r54659

**Published:** 2023-02-16

**Authors:** Ertan Mahir Korkmaz

**Affiliations:** 1 Department of Molecular Biology and Genetics, Sivas Cumhuriyet University, Sivas, Turkey

The manuscript focuses on the genome assembly of Lasioglossum lativentre. The genome sequence data was constructed using Pacific Biosciences HiFi circular consensus and 10X Genomics read cloud sequencing libraries. The quality of this assembled genome seems to be acceptable based on the number and N50 values of the obtained scaffolds and contigs and completeness percent coming from BUSCO results. In my opinion, the authors should add a single and clear aim paragraph to the end of the section of "Background" describing the reason why the authors have sequenced and assembled this bee genome.

Are sufficient details of methods and materials provided to allow replication by others?

Yes

Is the rationale for creating the dataset(s) clearly described?

Yes

Are the datasets clearly presented in a useable and accessible format?

Yes

Are the protocols appropriate and is the work technically sound?

Yes

Reviewer Expertise:

insect mitogenomics and mitotranscriptomics, insect genome evolution, Hymenoptera

I confirm that I have read this submission and believe that I have an appropriate level of expertise to confirm that it is of an acceptable scientific standard.

# Peer review: Reviewer response for version 1

**DOI:** 10.21956/wellcomeopenres.19593.r54656

**Published:** 2023-02-09

**Authors:** Hidemasa Bono

**Affiliations:** 1 Laboratory of Genome Informatics, Graduate School of Integrated Sciences for Life, Hiroshima University, Higashihiroshima, Japan

Authors present the genome sequencing of the furry-claspered furrow bee, *Lasioglossum lativentre*. The methods were concisely described, but the software used in the analysis were sufficiently summarized as Table 3 with versions and sources. Data described in the manuscript were fully opened from the public repository. 

 Only the genome sequence was reported in this manuscript. This reviewer cannot see why 'iyLasLatv1'(female) and 'iyLasLatv2' (male) were used.

 Authors used iyLasLatv1 in genome sequencing, but the name of assembly identifier is 'iyLasLatv2'.

Are sufficient details of methods and materials provided to allow replication by others?

Partly

Is the rationale for creating the dataset(s) clearly described?

Yes

Are the datasets clearly presented in a useable and accessible format?

Yes

Are the protocols appropriate and is the work technically sound?

Yes

Reviewer Expertise:

Genome biology

I confirm that I have read this submission and believe that I have an appropriate level of expertise to confirm that it is of an acceptable scientific standard, however I have significant reservations, as outlined above.

# Peer review: Reviewer response for version 1

**DOI:** 10.21956/wellcomeopenres.19593.r53576

**Published:** 2023-02-01

**Authors:** Alexandre R. Paschoal

**Affiliations:** 1 Department of Computer Science, Federal University of Technology-Paraná (UTFPR), Cornélio Procópio, Brazil

The authors provide a brief data genome assembly and BUSCO annotation on the Lasioglossum lativentre genome. The manuscript is well described in a summary report. My comments are to help to address more detail in this work. I hope can contribute to improving it.

 1) Could authors clarify where the genome assembly data is?

 The authors mention the Accession number PRJEB46299 at the ENA.

 We have only fastaq for the ID: PRJEB46259. Next, I found the https://projects.ensembl.org/darwin-tree-of-life/ with the Lasioglossum lativentre data. But only soft masked genome is available. Why? and no masked and hard masked?

 2) Could you do a GFF and FASTA for Noncoding RNAs in https://projects.ensembl.org/darwin-tree-of-life/ webpage?

 Do the same in Ensembl FTP, please: https://ftp.ensembl.org/pub/rapid-release/species/Lasioglossum_lativentre/GCA_916610255.1/ensembl/geneset/2022_02/

 I feel a lack of ncrna file.

 3) I feel a lack of basic annotation statistics and discussion:

- Repeat elements, particularly transposable elements.
- Noncoding RNAs genes.

4) Considering item 4 and coding genes, I feel a lack of comparison with other "related" genomes, for example:

 a) Lasioglossum albipes genome ^2^.

 b) In particular to Frieseomelitta varia, a genome I have been in the consortium, and I am curious to see the comparison ^1^.

 c) With model insects like: D. melanogaster, Apis mellifera and Nasonia vitripennis.

Are sufficient details of methods and materials provided to allow replication by others?

Yes

Is the rationale for creating the dataset(s) clearly described?

Yes

Are the datasets clearly presented in a useable and accessible format?

Partly

Are the protocols appropriate and is the work technically sound?

Yes

Reviewer Expertise:

Bioinformatics

I confirm that I have read this submission and believe that I have an appropriate level of expertise to confirm that it is of an acceptable scientific standard.

## References

[1] : The nuclear and mitochondrial genomes of Frieseomelitta varia - a highly eusocial stingless bee (Meliponini) with a permanently sterile worker caste. *BMC Genomics* .2020;21(1) : 10.1186/s12864-020-06784-8 386 10.1186/s12864-020-06784-8 32493270PMC7268684

[2] : Social evolution. Genomic signatures of evolutionary transitions from solitary to group living. *Science* .2015;348(6239) : 10.1126/science.aaa4788 1139-43 10.1126/science.aaa4788 25977371PMC5471836
